# Supplementary material for: Understanding Marine Biodegradation of Bio-Based Oligoesters and Plasticizers
Source: Polymers (Basel). 2023 Mar 20;15(6):1536. doi: 10.3390/polym15061536 (PMC10054732; doi:10.3390/polym15061536)
Supplement: Supplementary file 1 [file polymers-15-01536-s001.zip › polymers-2244074-supplementary.pdf]

Understanding marine biodegradation of biobased oligoesters and plasticizers

Federico Zappaterra [1], Monia Renzi[2], Manuela Piccardo[2], Mariachiara Spennato[1], Fioretta Asaro[1], Martino Di Serio[3], Rosa Turco[3,4], Anamaria Todea[1*], Lucia Gardossi[1]

*Correspondence: atodea@units.it

Supplementary material

Figure S1a. Schematic representation of synthesis of the products obtainable from the ring opening of epoxidized triolein with 1,4-butandiol 3

Figure S1b. Schematic representation of synthesis of the products obtainable from the ring opening of epoxidized triolein with sorbitol 4

Figure S2. ESI-MS spectrum of the oligoesters synthesized starting from 1,4-BDO and AA at 70°C, in solvent-less system, using covalently immobilized CalB. 5

Figure S3. Enlarged ^{1}H-NMR spectrum of poly(1,4-butylene adipate) in DMSO-d_6........ 5

Figure S4. FT-IR spectra of the epoxidized oil, 1,4-butandiol, product ECO-SRB, Product ECO-BDO. 6

Figure S5. ^{1}H NMR spectrum of the product of the epoxidation reaction of oleic acid catalysed by Novozyme 435 in 2h 7

Figure S6. Enlarged ^{1}H NMR spectrum of the product obtained after 2 h of epoxidation of linoleic acid catalysed by 450 U/$g_{substrate}$ of lipase 7

Figure S7. Enlarged ^{1}H NMR spectrum of the product obtained after 3 h of epoxidation of linolenic acid catalysed by 450 U/g substrate of lipase 8

Figure S8. GC-MS chromatograms of a) oleic acid b) reaction mixture after 2 h in which is evident the complete conversion into epoxy product (9,10-epoxistearic acid). 8

Figure S9. GC-MS chromatogram of a) linoleic acid, b) reaction mixture after 3 h. 9

Figure S10. GC-MS chromatogram of a) linolenic acid, b) reaction mixture. 9

Figure S11. The thermogram of co-oligoester containing AA-BDO units 10

Figure S12. The thermogram of sample ECOSORB........ 10

Figure S13. The thermogram of sample ESOBDO 10

Figure S14. Differential scanning calorimetry characterization (DSC) curves of the $(BDO\text{-}AA)_n$ sample, cooling (red), 2nd heating cycle (blue)........ 11

Figure S15. Differential scanning calorimetry characterization (DSC) curves of the $(Gly\text{-}AZA)_n$ sample, cooling (red), 2nd heating cycle (blue)........ 11

Figure S16. Differential scanning calorimetry characterization (DSC) curves of the Esobdo sample, cooling (red), 2nd heating cycle (blue)........ 12

Figure S17. Differential scanning calorimetry characterization (DSC) curves of the Ecosorb sample, cooling (red), 2nd heating cycle (blue) 12

Figure S18. Differential scanning calorimetry characterization (DSC) curves of the EPX_OIL_C sample, cooling (red), 2nd heating cycle (blue) 13

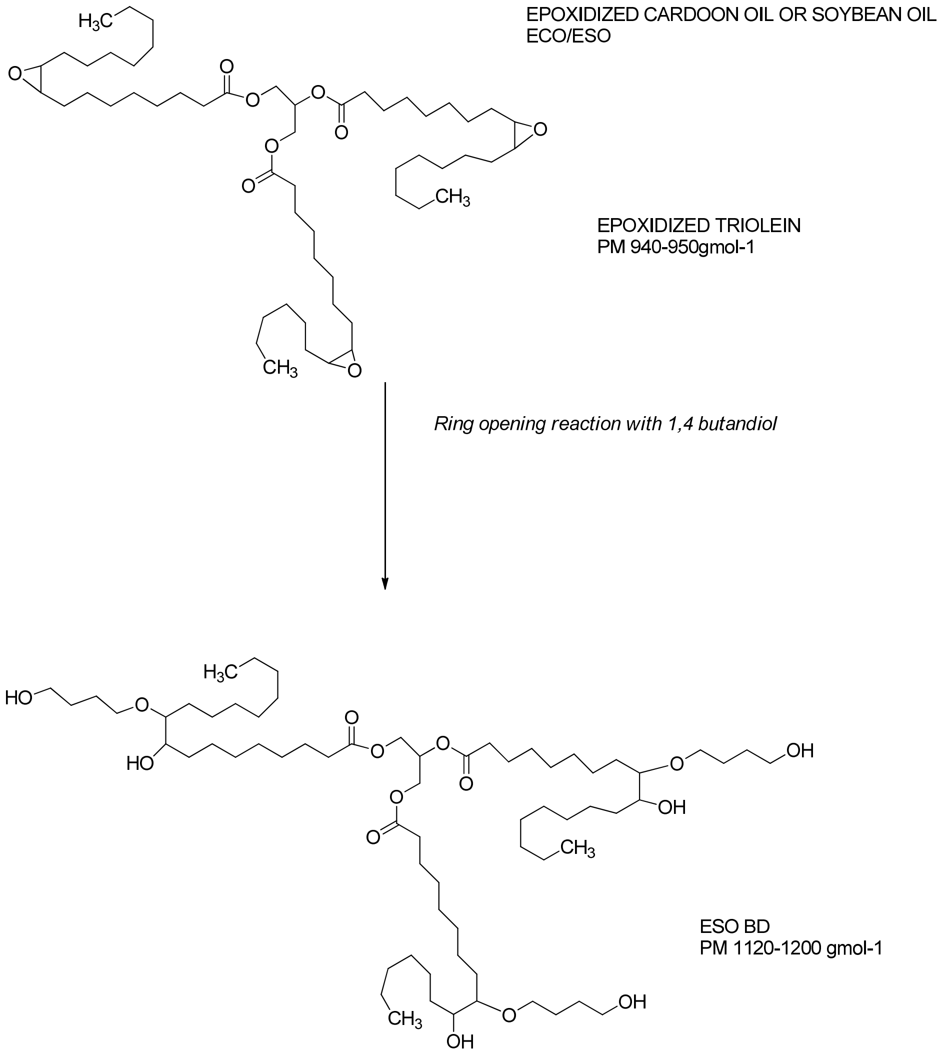

Figure S1a. Schematic representation of synthesis of the products obtainable from the ring opening of epoxidized triolein with 1,4-butandiol

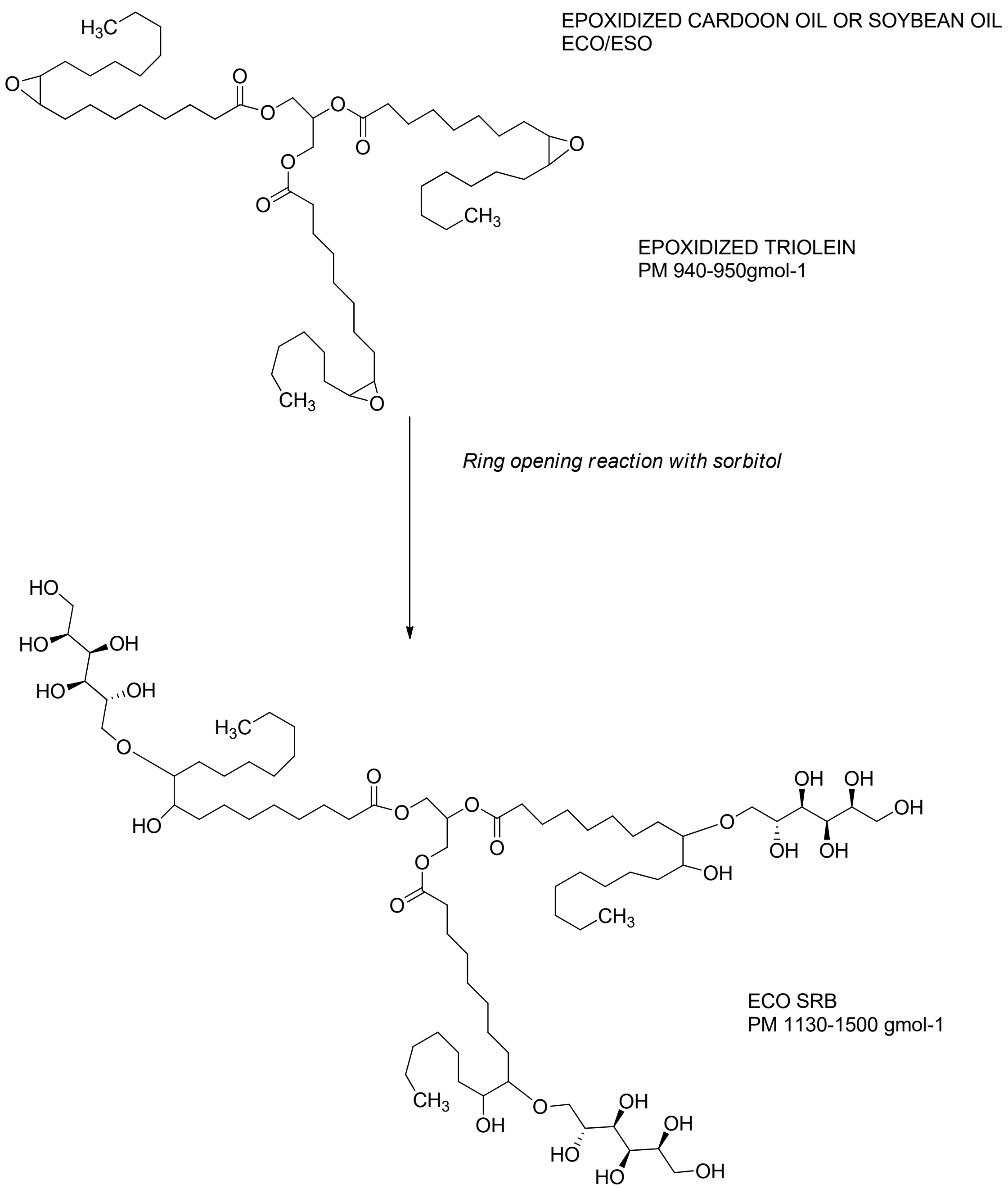

Figure S1b. Schematic representation of synthesis of the products obtainable from the ring opening of epoxidized triolein with sorbitol

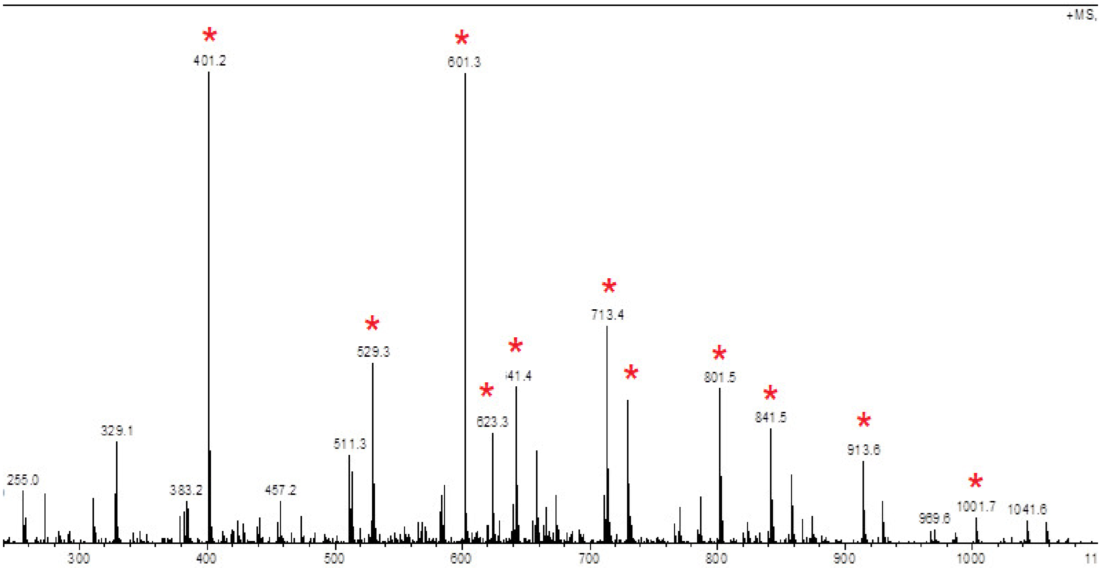

Figure S2. ESI-MS spectrum of the oligoesters synthesized starting from 1,4-BDO and AA at 70°C, in solvent-less system, using covalently immobilized CalB.

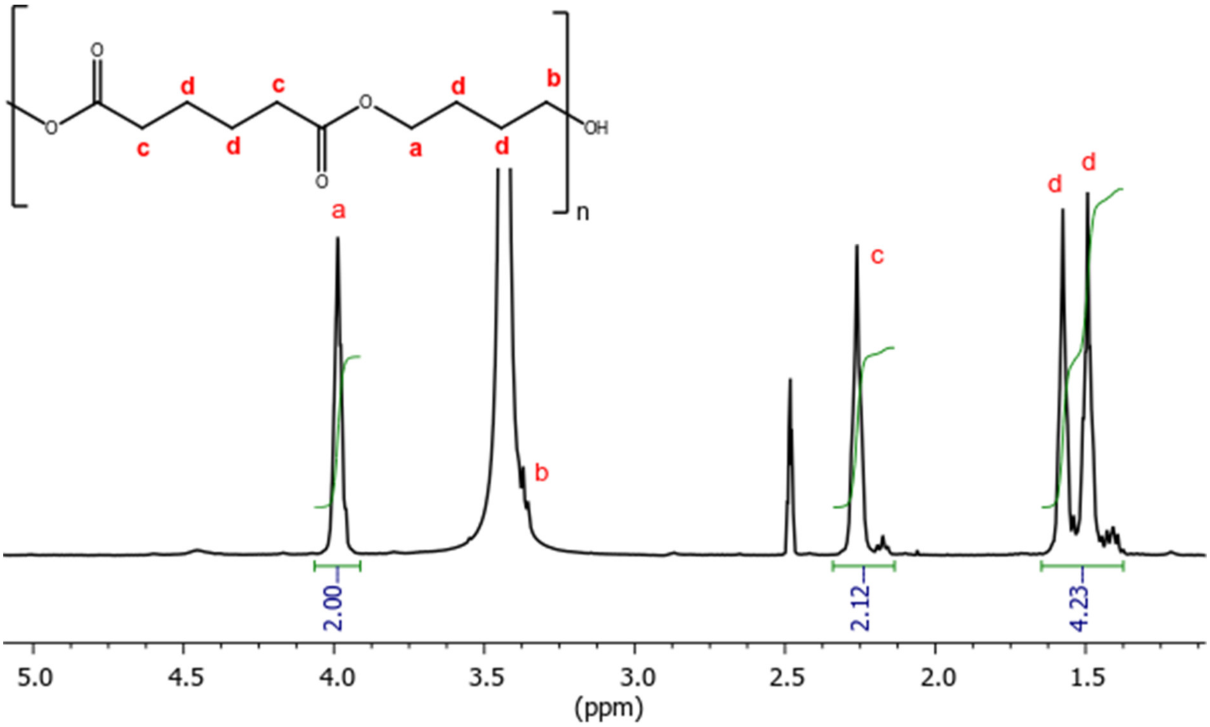

Figure S3. Enlarged ^{1}H-NMR spectrum of poly(1,4-butylene adipate) in DMSO-d_6, with assignment of the signals according to [1].

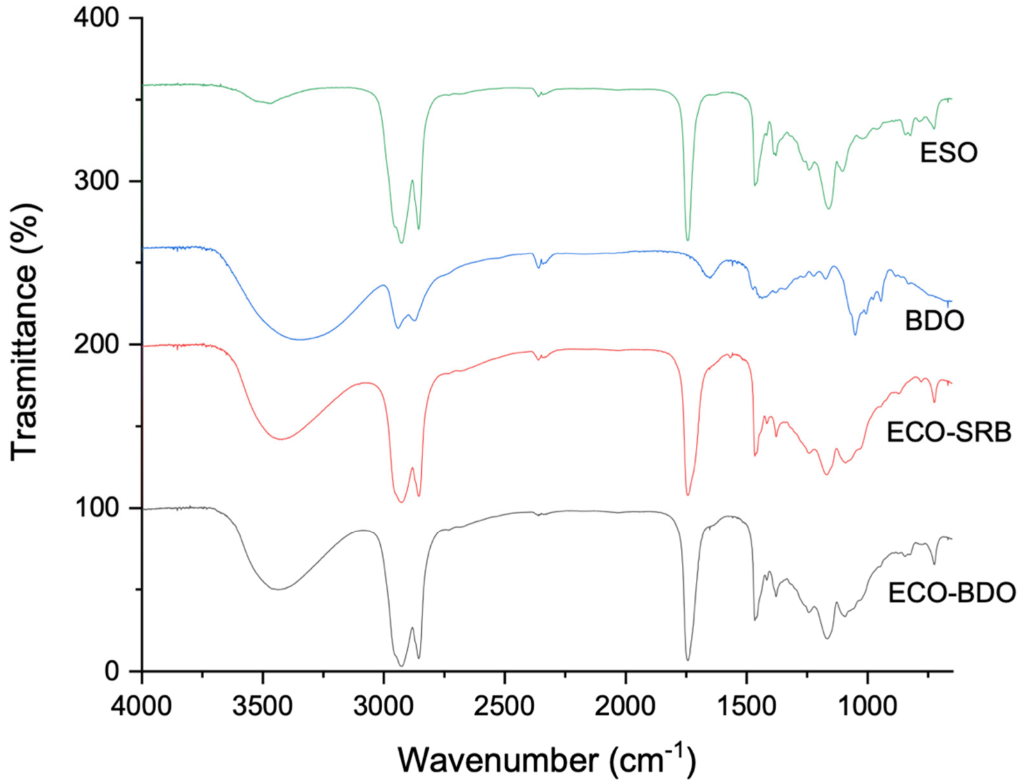

Figure S4. FT-IR spectra of the epoxidized oil (green), 1,4-butandiol (blue), product ECO-SRB (red), Product ECO-BDO (black).

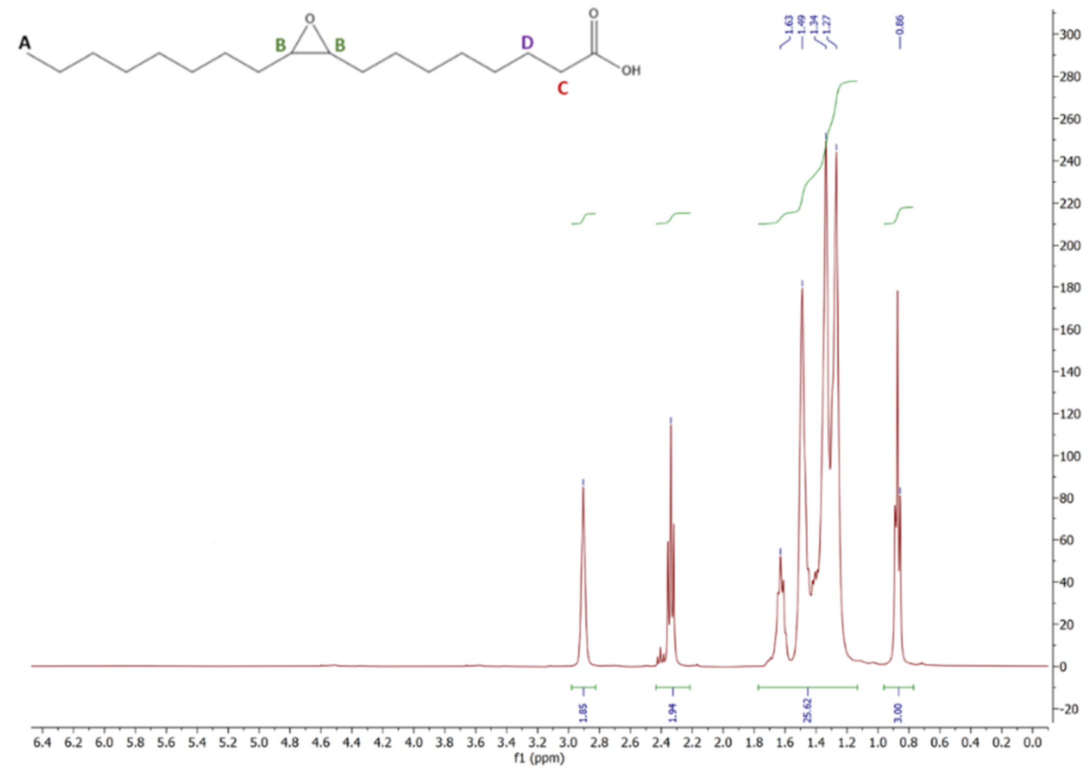

Figure S5. ^{1}H NMR spectrum of the product of the epoxidation reaction of oleic acid catalysed by Novozyme 435 in 2h. δ=0.89 ppm, -CH_3 (A); δ=1.36ppm -CH_2- of chain, δ=1.63ppm -CH_2- (D); δ:2,64ppm -CH_2- (C); δ:2.90ppm -CH- (B). Assignment of the signals according to [2].

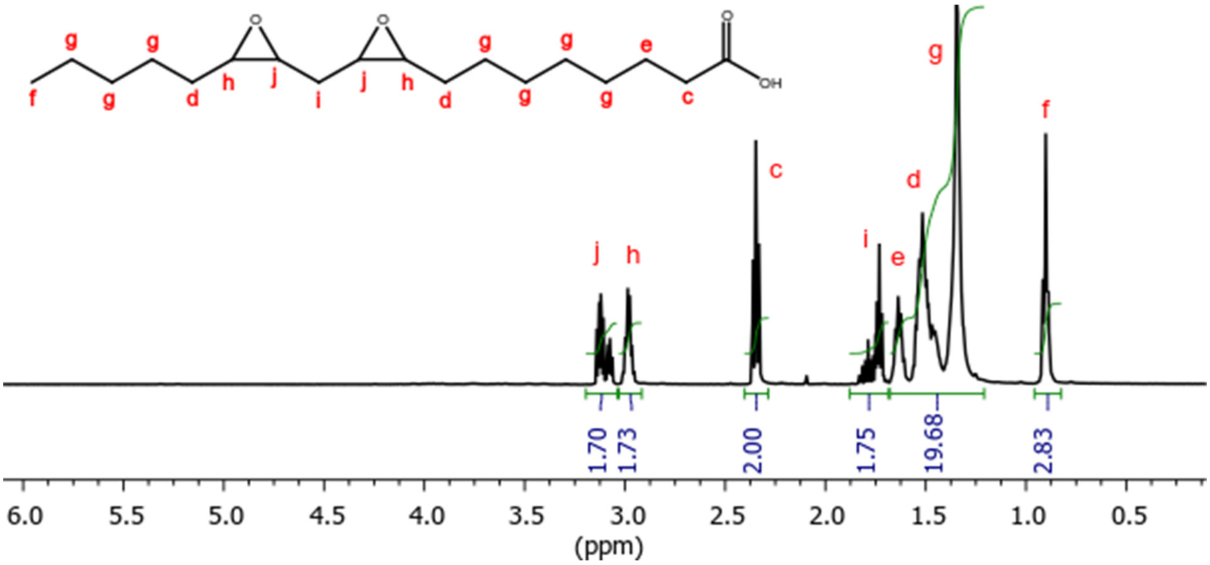

Figure S6. Enlarged ^{1}H NMR spectrum of the product obtained after 2 h of epoxidation of linoleic acid catalysed by 450 U/$g_{substrate}$ of lipase, with assignment of the signals according to [3].-

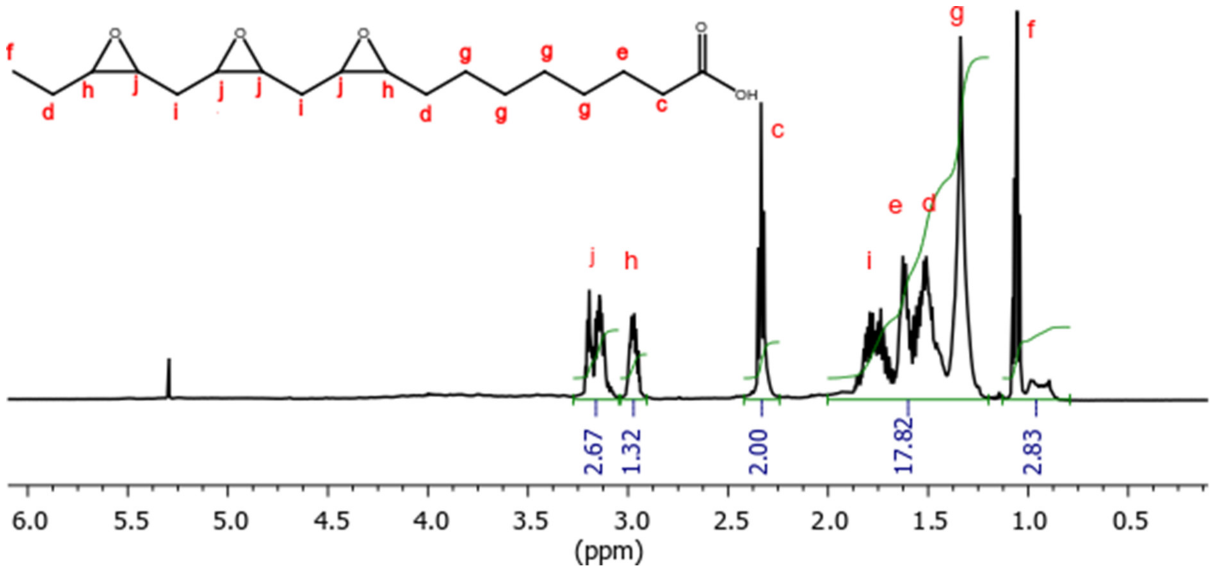

Figure S7. Enlarged ^{1}H NMR spectrum of the product obtained after 3 h of epoxidation of linolenic acid catalysed by 450 U/g substrate of lipase, with assignment of the signals according to [3].

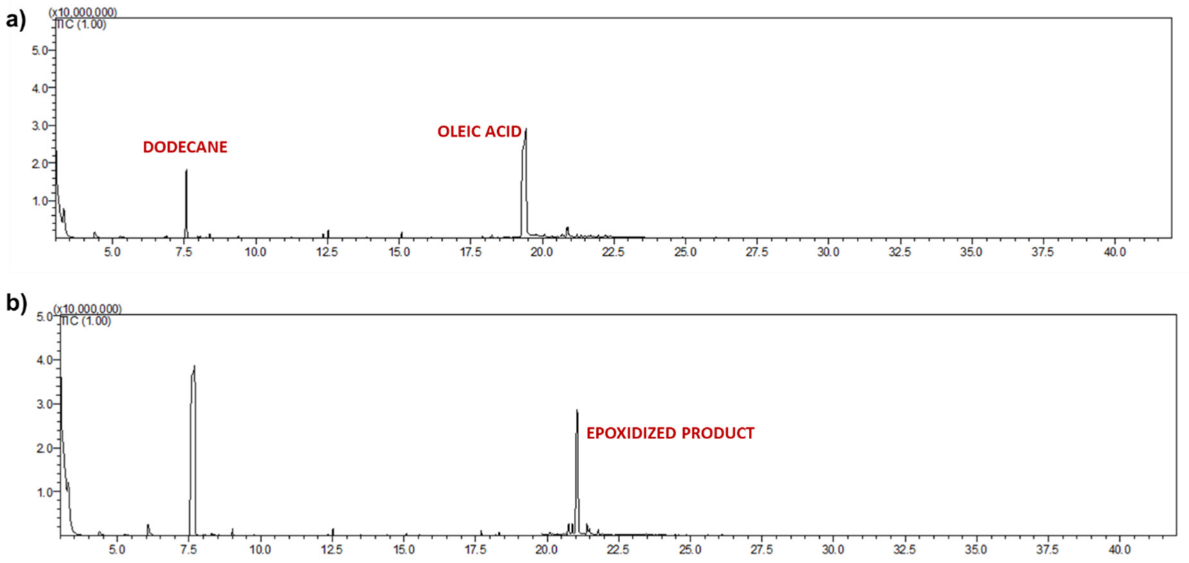

Figure S8. GC-MS chromatograms of a) oleic acid b) reaction mixture after 2 h in which is evident the complete conversion into epoxy product (9,10-epoxistearic acid).

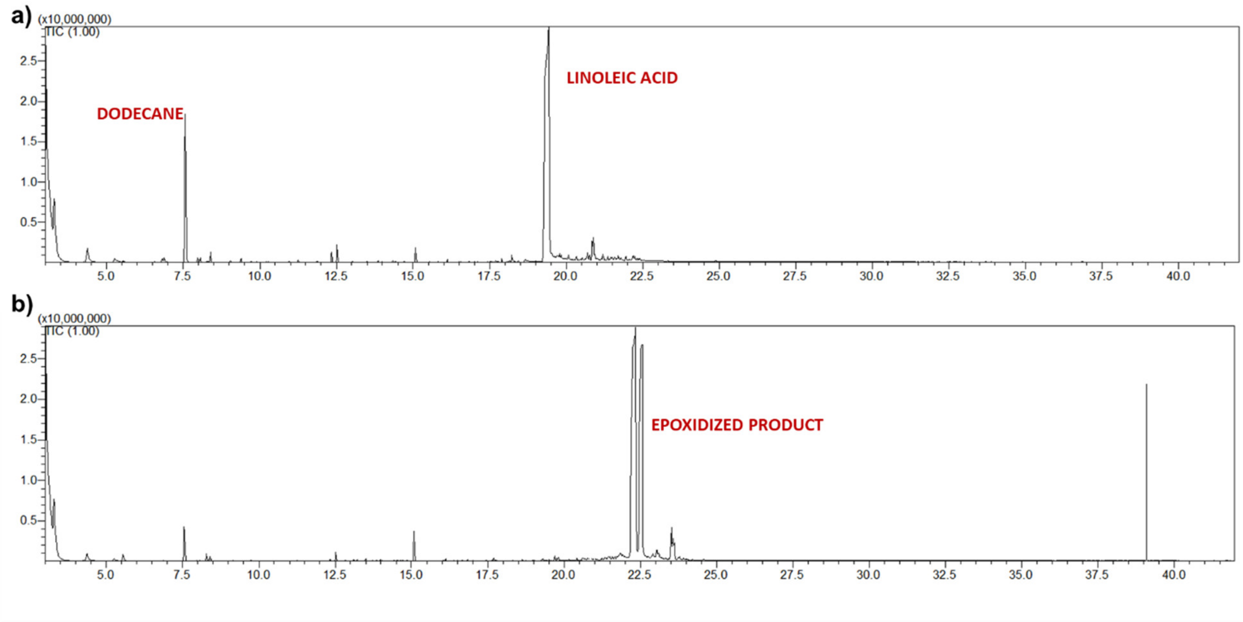

Figure S9. GC-MS chromatogram of a) linoleic acid, b) reaction mixture after 3 h.

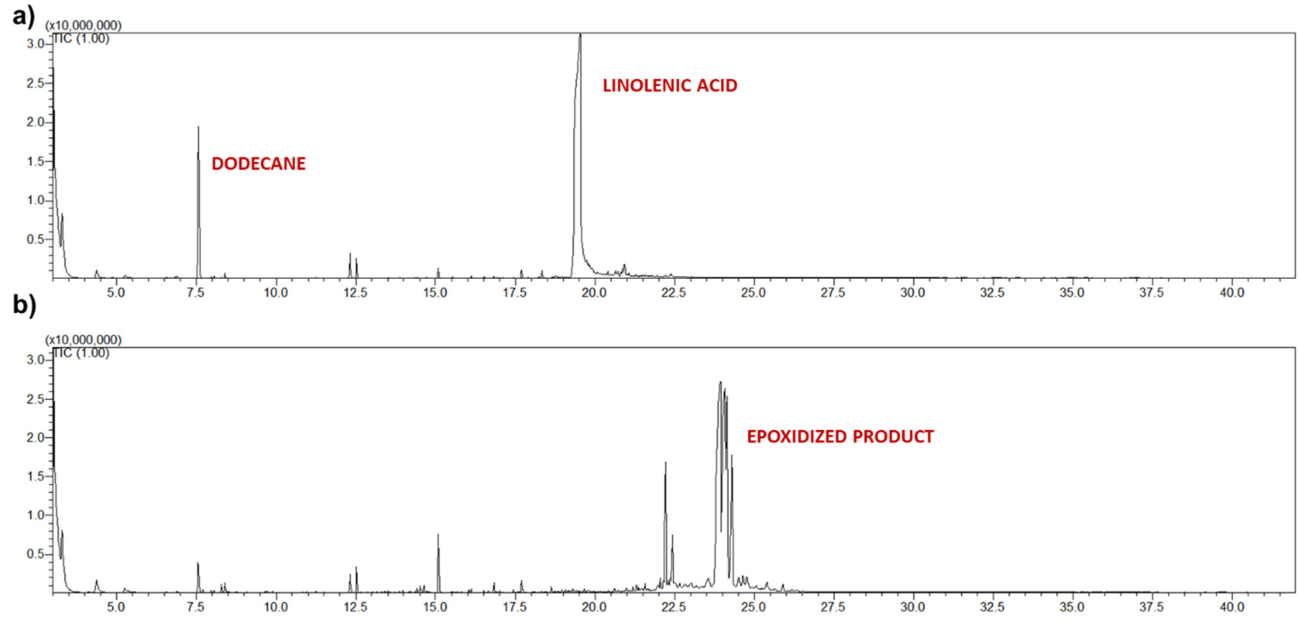

Figure S10. GC-MS chromatogram of a) linolenic acid, b) reaction mixture.

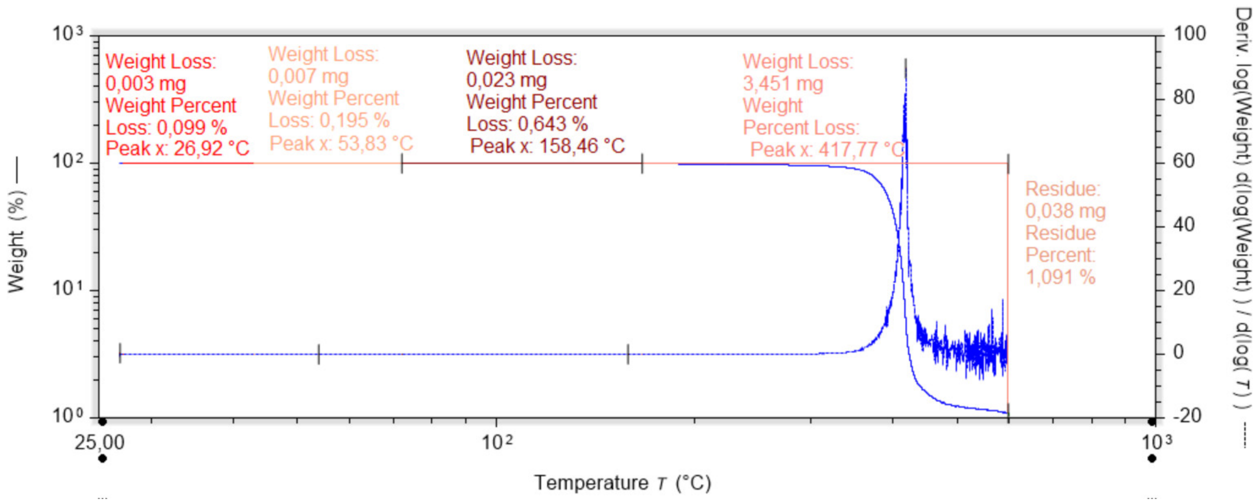

Figure S11. The thermogram of co-oligoester containing AA-BDO units

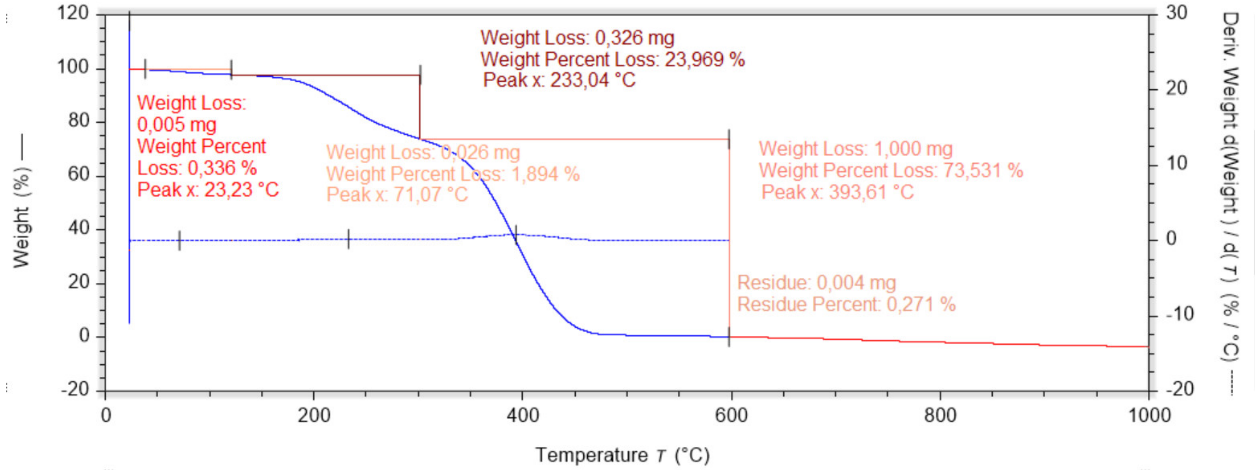

Figure S12. The thermogram of sample ECOSORB

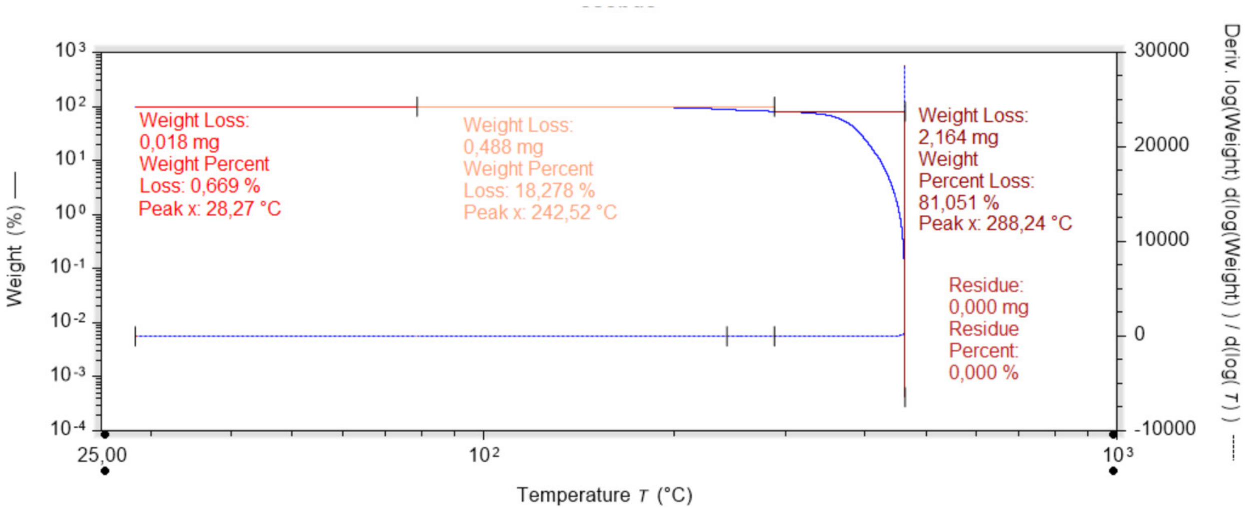

Figure S13. The thermogram of sample ESOBDO

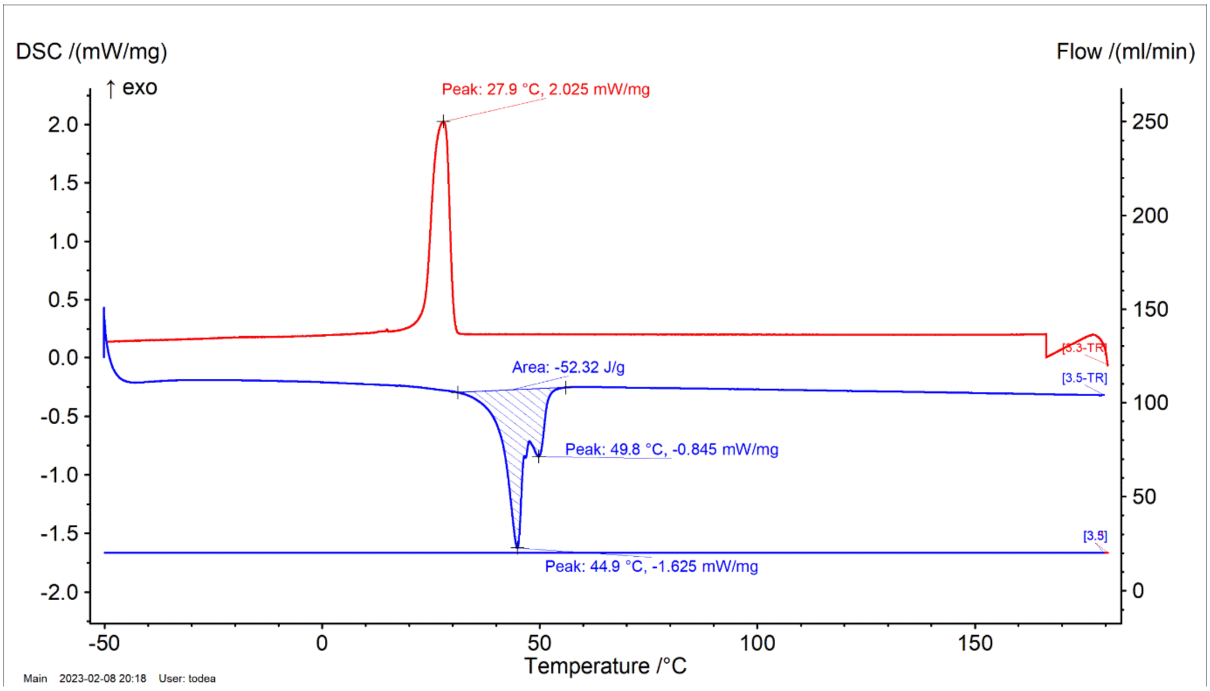

Figure S14. Differential scanning calorimetry characterization (DSC) curves of the $(BDO\text{-}AA)_n$ sample, cooling (red), 2nd heating cycle (blue)

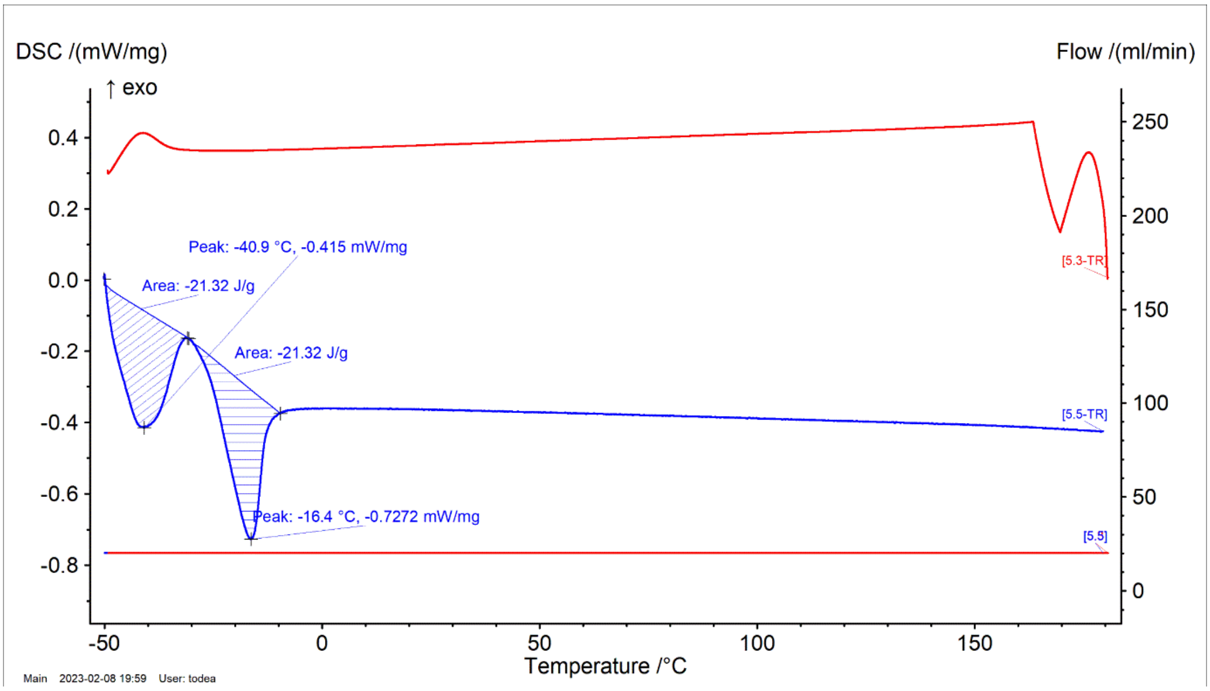

Figure S15. Differential scanning calorimetry characterization (DSC) curves of the $(Gly\text{-}AZA)_n$ sample, cooling (red), 2nd heating cycle (blue)

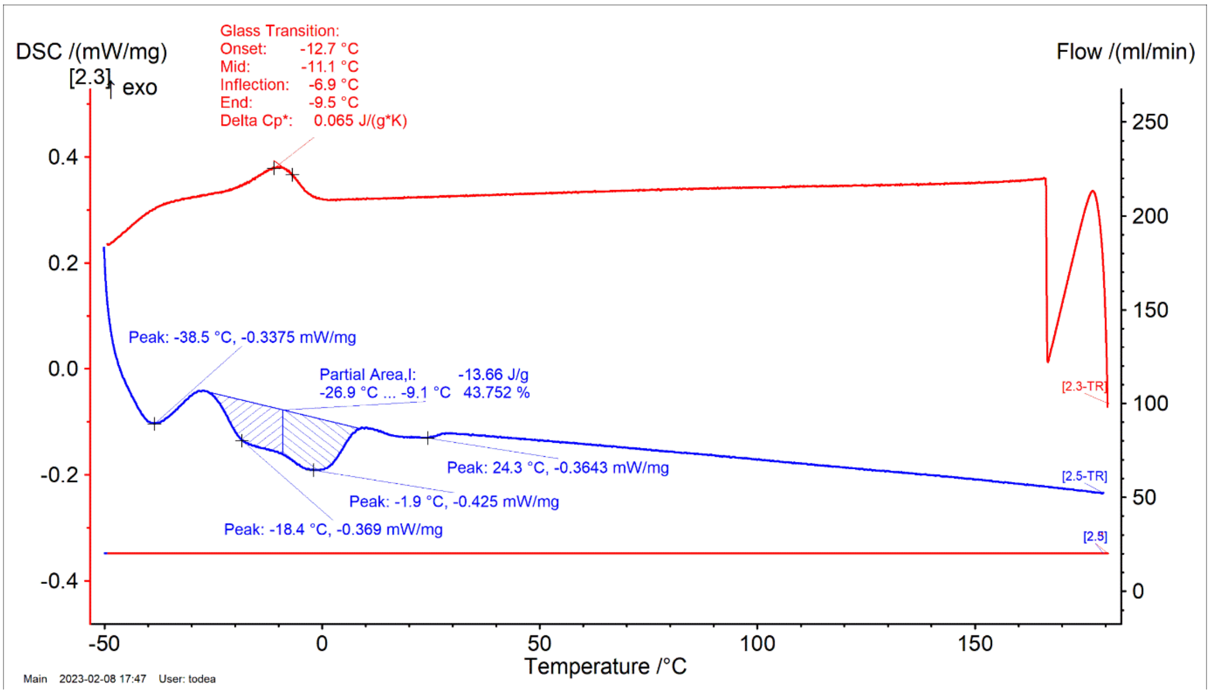

Figure S16. Differential scanning calorimetry characterization (DSC) curves of the Esobdo sample, cooling (red), 2nd heating cycle (blue)

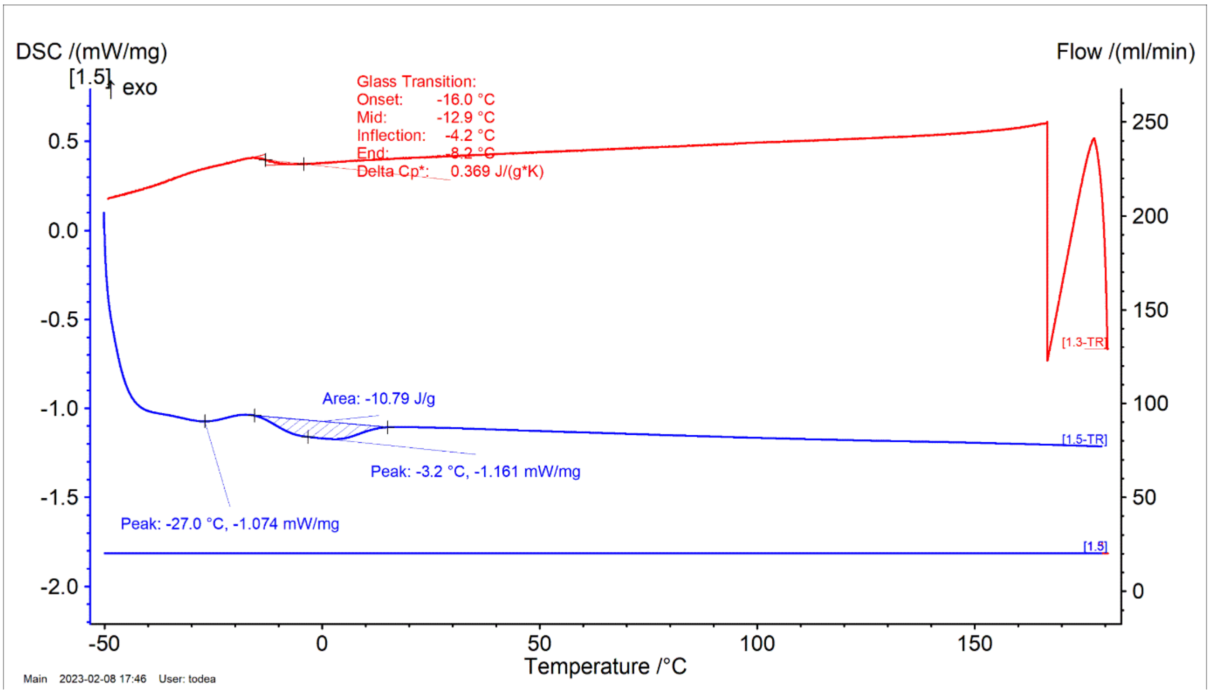

Figure S17. Differential scanning calorimetry characterization (DSC) curves of the Ecosorb sample, cooling (red), 2nd heating cycle (blue)

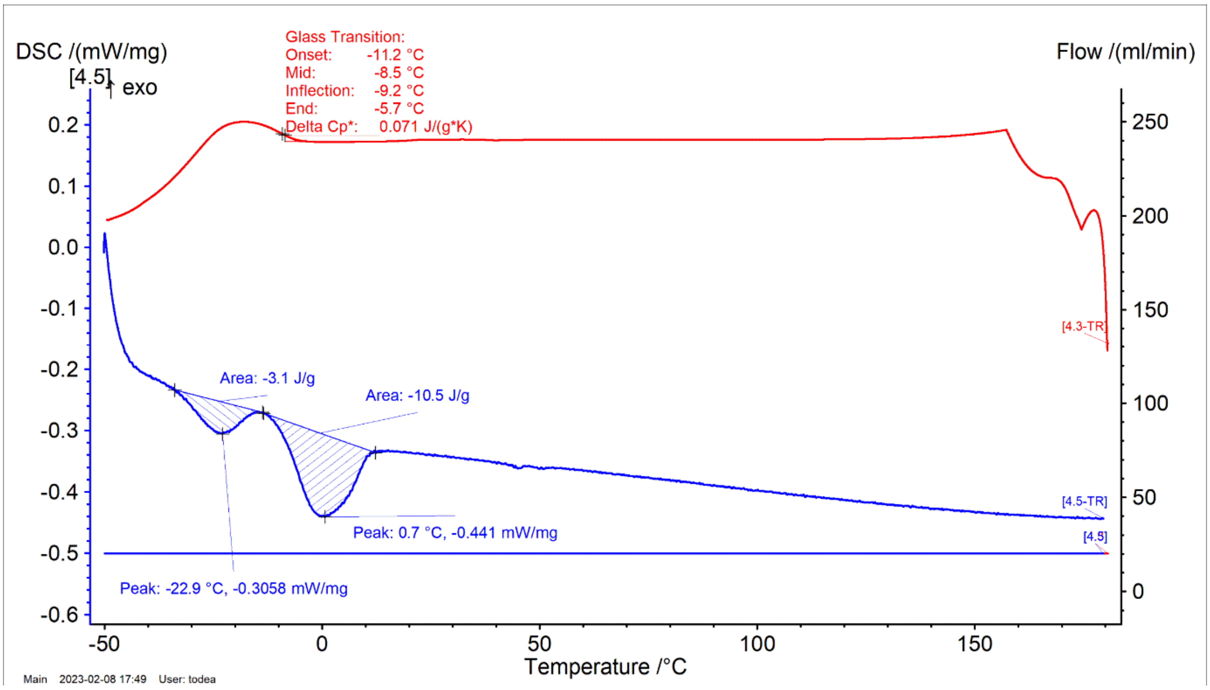

Figure S18. Differential scanning calorimetry characterization (DSC) curves of the EPX_OIL_C sample, cooling (red), 2nd heating cycle (blue)

SI references

1. Weinberger, S.; Pellis, A.; Comerford, J. W.; Farmer, T. J.; Guebitz, G. M. Efficient Physisorption of Candida Antarctica Lipase B on Polypropylene Beads and Application for Polyester Synthesis. *Catalysts* **2018**, *8* (9). https://doi.org/10.3390/catal8090369.
2. Kaur, A.; Bhardwaj, N.; Kaur, A.; Abida, K.; Nagaraja, T. P.; Ali, A.; Prakash, R. Proton Nuclear Magnetic Resonance-Based Method for the Quantification of Epoxidized Methyl Oleate. *JAOCS, J. Am. Oil Chem. Soc.* **2021**, *98* (2), 139–147. https://doi.org/10.1002/aocs.12439.
3. Xia, W.; Budge, S. M.; Lumsden, M. D. 1H-NMR Characterization of Epoxides Derived from Polyunsaturated Fatty Acids. *JAOCS, J. Am. Oil Chem. Soc.* **2016**, *93* (4), 467–478. https://doi.org/10.1007/s11746-016-2800-2.
